# Quality assessment of malaria microscopic diagnosis at the Aristide Le Dantec University Hospital of Dakar, Senegal, in 2020

**DOI:** 10.1186/s13104-023-06571-0

**Published:** 2024-03-09

**Authors:** Mamane N. Garba, Awa B. Dème, Khadim Diongue, Younousse Diédhiou, Amadou M. Mbaye, NDèye M. Dia, N’Dèye A. Seck, Daba Zoumarou, Lamine Ndiaye, Mamadou S. Yade, Baba Dièye, Aita Sène, Abdoulaye Tine, Mariama Touré, Nogaye Gadiaga, Awa Fall, Bassirou Ngom, Djiby Sow, Aliou Ndiaye, Astou Keita, Mame F. Ndiaye, Jules F. Gomis, Nana F. Diop, Guète Diallo, Ibrahima M. Ndiaye, Elhadj M. Ba, Omar Bitèye, Cheikh Ndiaye, Fama S. D. Mbodji, Pape O. Ndiaye, Tolla Ndiaye, Amy Gaye, Mouhamad Sy, Yaye D. Ndiaye, Mame C. Seck, Mouhamadou Ndiaye, Aida S. Badiane, Mamadou A. Diallo, Daouda Ndiaye

**Affiliations:** 1 International Research Training Center on Genomics and Health Surveillance (CIGASS), Dakar, Senegal; 2 Laboratoire de Parasitologie-Mycologie, Université Cheikh Anta Diop de Dakar (UCAD), Hôpital Aristide Le Dantec, Dakar, Senegal

**Keywords:** *Plasmodium*, Diagnosis, Sensitivity, Specificity, Senegal

## Abstract

**Background:**

Following WHO guidelines, microscopy is the gold standard for malaria diagnosis in endemic countries. The Parasitology-Mycology laboratory (LPM) is the National Reference Laboratory and is currently undergoing ISO 15189 accreditation. In this context, we assessed the performance of the laboratory by confirming the reliability and the accuracy of results obtained in accordance with the requirements of the ISO 15189 standards. This study aimed to verify the method of microscopic diagnosis of malaria at the LPM, in the Aristide Le Dantec hospital (HALD) in Dakar, Senegal.

**Methods:**

This is a validation/verification study conducted from June to August 2020. Twenty (20) microscopic slides of thick/thin blood smear with known parasite densities (PD) selected from the Cheick Anta Diop University malaria slide bank in Dakar were used for this assessment. Six (6) were used to assess microscopists’ ability to determine PD and fourteen (14) slides were used for detection (positive vs negative) and identification of parasites. Four (4) LPM-HALD microscopists read and recorded their results on prepared sheets. Data analysis was done with Microsoft Excel 2010 software.

**Results:**

A minimum threshold of 50% concordance was used for comparison. Of the twenty (20) slides read, 100% concordance was obtained on eight (8) detection (positive vs negative) slides. Four (4) out of the six (6) parasite density evaluation slides obtained a concordance of less than 50%. Thirteen (13) out of the fourteen (14) identification slides obtained a concordance greater than 50%. Only one (1) identification slide obtained zero agreement from the microscopists. For species identification a concordance greater than 80% was noted and the microscopists obtained scores between 0.20 and 0.4 on a scale of 0 to 1 for parasite density reading. The microscopists obtained 100% precision, sensitivity, specificity and both negative and positive predictive values.

**Conclusion:**

This work demonstrated that the microscopic method of malaria diagnosis used in the LPM/HALD is in accordance with the requirements of WHO and ISO 15189. Further training of microscopists may be needed to maintain competency.

## Background

In Senegal, the National Malaria Control Program (NMCP) and its partners have adopted control strategies for the elimination of malaria. They contributed to registering a significant reduction of more than 50% of the disease burden between 2009 and 2015 with a parasite prevalence which decreased from 3 to 1.2% and mortality from 72 to 33 per 100,000 children less than 5 years at risk [1–3].

These strategies include laboratory diagnosis, which reflects the WHO recommendation that only confirmed cases should be treated with ACTs. In endemic areas, RDTs and light microscopy are the most widely used and the latter constitutes the reference standard according to WHO recommendations [4–8].

Microscopy is available in intermediate, central, and peripheral health facilities or rural health centers. The quality of microscopic diagnosis is essential to guarantee adequate treatment to maintain this trend of reduction in morbidity and mortality linked to malaria. The effectiveness of malaria microscopic diagnosis remains dependent on maintaining a high level of staff skills and performance, the availability of good quality reagents and equipment at all levels, and regular internal and external evaluations [5–7, 9–12].

The Parasitology and Mycology Laboratory of the Aristide Le Dantec Hospital (LPM/HALD) is enrolled in an accreditation process through the West African Society of Accreditation and Certification according to ISO 15189 version 2012 [13].Using the microscopic method of malaria diagnosis, it must demonstrate the performance of the laboratory system, confirm the reliability and accuracy of the results obtained and ensure continuous improvement of the Quality Management System.

Indeed, the ISO 15189 Standard is an accreditation standard which presents general requirements concerning the quality and competence of Biomedical Laboratories. Among the requirements of this standard for accreditation is the verification and the validation of the methods used for diagnosis. Here, we assessed the reliability and accuracy of microscopy results obtained in accordance with the requirements from the ISO 15189 standard [13–19].

The objectives were (1) to determine the concordance of results obtained between microscopists, (2) to measure the qualitative indicators of malaria diagnosis (identification, parasite density), and (3) to estimate the sensitivity and specificity as well as the predictive values of the different microscopists.

## Methods

The Laboratory of Parasitology and Mycology at Aristide Le Dantec Hospital in Dakar, Senegal, is the National Reference Laboratory for malaria in Senegal and as such supports the NMCP in the microscopic diagnosis of malaria. This laboratory is also involved, under the agreement of WHO and the NMCP, in the training and accreditation of African experts in microscopy.

### Type and period of study

This is a verification assessment study conducted from June to August 2020.

### Study sample

Slides of thick blood smears and thin smears made and stained by LPM-HALD with known parasite densities were chosen from the slide bank of Cheick Anta Diop University (UCAD). One (1) of these slides was repeated three times. The slides were validated by the WHO Level 1 expert microscopists in Senegal and by real-time PCR at UCAD.

### Sample size

Twenty (20) slides were examined in accordance with WHO recommendations as part of microscopist certification using the WHO competence levels and criteria (Table 1) [2, 3].For the general characteristics of the evaluation slides (Table 2), a total of twenty (20) slides were read by four (4) different microscopists. Among these twenty (20) slides, six (6/20) positive thick blood smears were used to assess the ability of the microscopists to determine the parasite density (PD) and the other fourteen (14/20) were used for parasite identification. Of these fourteen (14), eight (8) were positive with at least one species of *Plasmodium* and six (6) were negative with no parasite.

**Table 1:** WHO competence levels and criteria

Competence level	Parasite detection (%)	Species identification (%)	Parasite density within 25% of the true count (%)
1	90–100	90–100	50–100
2	80–89	80–89	40–49
3	70–79	70–79	30–39
4	0–69	0–69	0–29

**Table 2:** General characteristics of the evaluation slides

Slide N﻿o	References			
	﻿Results	﻿Species/PD (number of parasites/μl)	PD Margins	
			Bottom margin	Top margin
1	Negative			
2	Positive	Pm		
3	Positive	Pf/175		
4	Positive	Po		
5	Negative			
6	Positive	Pf/366	275	458
7	Positive	Pm		
8	Negative			
9	Positive	763	572	954
10	Positive	Pf + Po		
11	Negative			
12	Positive	Pf/175		
13	Positive	Pf/1483	1112	1854
14	Positive	Pm		
15	Negative			
16	Negative			
17	Positive	Pf/60500	45,375	75,625
18	Positive	Pv		
19	Positive	Pf/2160	1620	2700
20	Positive	Pf/175	131	219

Margin: 25% around the true value of the reference PD

*Pf* *Plasmodium falciparum*, *Pm* *Plasmodium malariae*, *Pv* *Plasmodium vivax*, *Po* *Plasmodium ovale*, *PD* parasite density

### Description of variables and data collection

Following the methods of the technical guide for accreditation, four (4) microscopists from LPM/HALD each read and recorded the results of the twenty (20) mixed thick/thin blood smear slides using the internal competency assessment form for malaria microscopists in Senegal (also called “collection sheet”) prepared by the slide bank at LPM-HALD. The aim was to identify *P. falciparum* and other species responsible for malaria after reading the slides using the 100 × oil immersion objective lens on the microscope. The determination of the parasite density on thin smear for positive slides was made according to the following formula [6, 13, 19]:

$$\mathrm{PD}=\frac{\mathrm{number\, of\, parasites\, counts }}{\mathrm{number\, of\, WBC\, counts }}\times 8000$$

*PD: Parasite Density: number of parasites/μl or mm^3^ of blood (corresponding to 8000 WBC).

*WBC: White Blood Cells (200 WBC for a thick blood smear with high PD and 500 WBC for a thick blood smear with low PD).

The determination of the scores obtained by the participants was carried out using the WHO method which corresponds to the number of correct results [identification and PD] on the total number of slides read [6, 11].

The determination of the concordance between microscopists was carried out according to the WHO method corresponding to the common results obtained by microscopists and which agree with the reference. The results were compared to an average concordance of 50% [7, 12].

### Inter-operator variability/concordance of a qualitative method

The inter-operator variability constitutes an indicator of the control of the realization of non-automated methods. The laboratory will be able to use the inter-operator variability and compare it to the intra-operator variability of a referent.

Another possibility to quantify the inter-operator variability will be the analysis of variance applied to the results obtained by the operators. Likewise, agreement can be used to measure inter-operator variability [13, 15].

ISO 5725 uses two terms “accuracy” and “precision” to describe the accuracy of a measurement method. “Trueness” refers to the closeness of agreement between the arithmetic mean of many test results and the true or accepted reference value. A qualitative method will be even more accurate if the values obtained are close to 100 when they are expressed in% [13, 16, 17].

### The precision

The precision is, according to ISO 3534–1, “the closeness of agreement between independent test results obtained under specified conditions.

The mathematical definition is:

$$Precision \left(\%\right)=\frac{\mathrm{TP}}{\mathrm{TP}+\mathrm{FP}}\times 100$$

### Accuracy

The accuracy is, according to ISO 3534-1, the closeness of agreement between the mean value obtained from a large series of test results and an accepted reference value. Microscopy performance measures the correctness of the results [accuracy of diagnosis and report] of the microscopist in everyday practice [12, 13, 18].

The mathematical definition is:

$$Accuracy \left(\%\right)=\frac{\mathrm{TP}+\mathrm{TN}}{\mathrm{TP}+\mathrm{TN}+\mathrm{FP}+\mathrm{FN}}\times 100$$

### Sensitivity (Se) and specificity (Sp)

According to the technical guide for accreditation of verification (scope A)/validation (scope B) of methods in medical biology, the concepts of sensitivity and specificity are used for dichotomous tests (yes or no, positive or negative, etc.). The sensitivity and specificity of a test give an appreciation of its intrinsic validity [13, 19].

Sensitivity is also called Fraction of True Positives which is the proportion of positive individuals detected by the test. In other words, sensitivity is a measure of how well the test performs when used on positive individuals. The test is perfect for positive individuals when the sensitivity is 1, equivalent to a random draw when the sensitivity is 0.5. If it is less than 0.5, the test is counter-performing. Thus, in the case of microscopy, it is the proportion of microscopists who detected positive slides knowing that the slides do indeed contain malaria parasites.

The mathematical definition is:

$$\mathrm{Sensitivity} \left(\%\right)=\frac{\mathrm{TP}}{\mathrm{TP}+\mathrm{FN}}\times 100$$

The Specificity is also called the True Negative Fraction which is the proportion of negative individuals effectively detected by the test. In other words, the specificity measures how well the test performs when used on negative individuals. The test is perfect for negative individuals when specificity is 1, equivalent to a random draw when specificity is 0.5. If it is less than 0.5, the test is counter-performing. Thus, for the case of microscopy, it is the proportion of microscopists who detected negative slides knowing that the slides do not contain a malaria parasite.

The mathematical definition is:

$$\mathrm{Specificity }\left(\%\right)=\frac{\mathrm{TN}}{\mathrm{TN}+\mathrm{FP}}\times 100$$

The positive predictive value (PPV) is the probability that the disease is present when the test is positive [13]. So, applied to microscopy, this is the probability that the parasite is present when the slide is positive.

The mathematical definition is:

$$\mathrm{PPV }\left(\%\right)=\frac{\mathrm{TP}}{\mathrm{TP}+\mathrm{FP}}\times 100$$

The negative predictive value (NPV) is the probability that the disease is not present when the test is negative [13]. In the case of microscopy, the NPV is the probability that the parasite is absent when the slide is negative.

The mathematical definition is:

$$\mathrm{NPV }\left(\%\right)=\frac{\mathrm{TN}}{\mathrm{TN}+\mathrm{FN}}\times 100$$

### Statistical analysis

Data was entered, coded, rechecked and analyzed using Microsoft Excel 2010 software. This verification assessment study was not intended or powered to conduct additional statistical analyses.

## Results

### Concordance of results obtained by microscopists

**Fig. 1:**
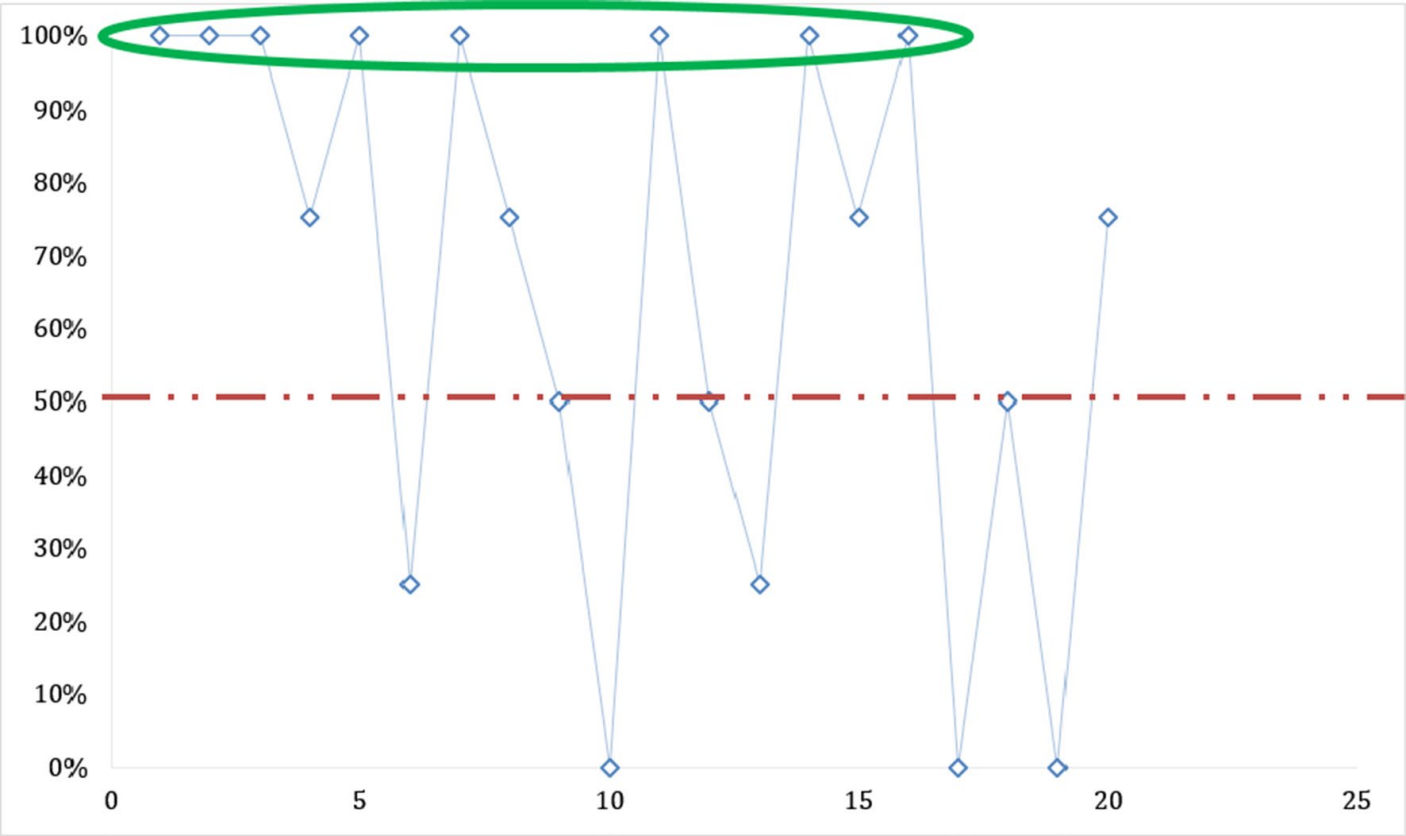
Concordance of results obtained between microscopists on the recognition of positive vs negative slides.  100% concordance of microscopists on the recognition of positive vs negative slides

**Fig. 2:**
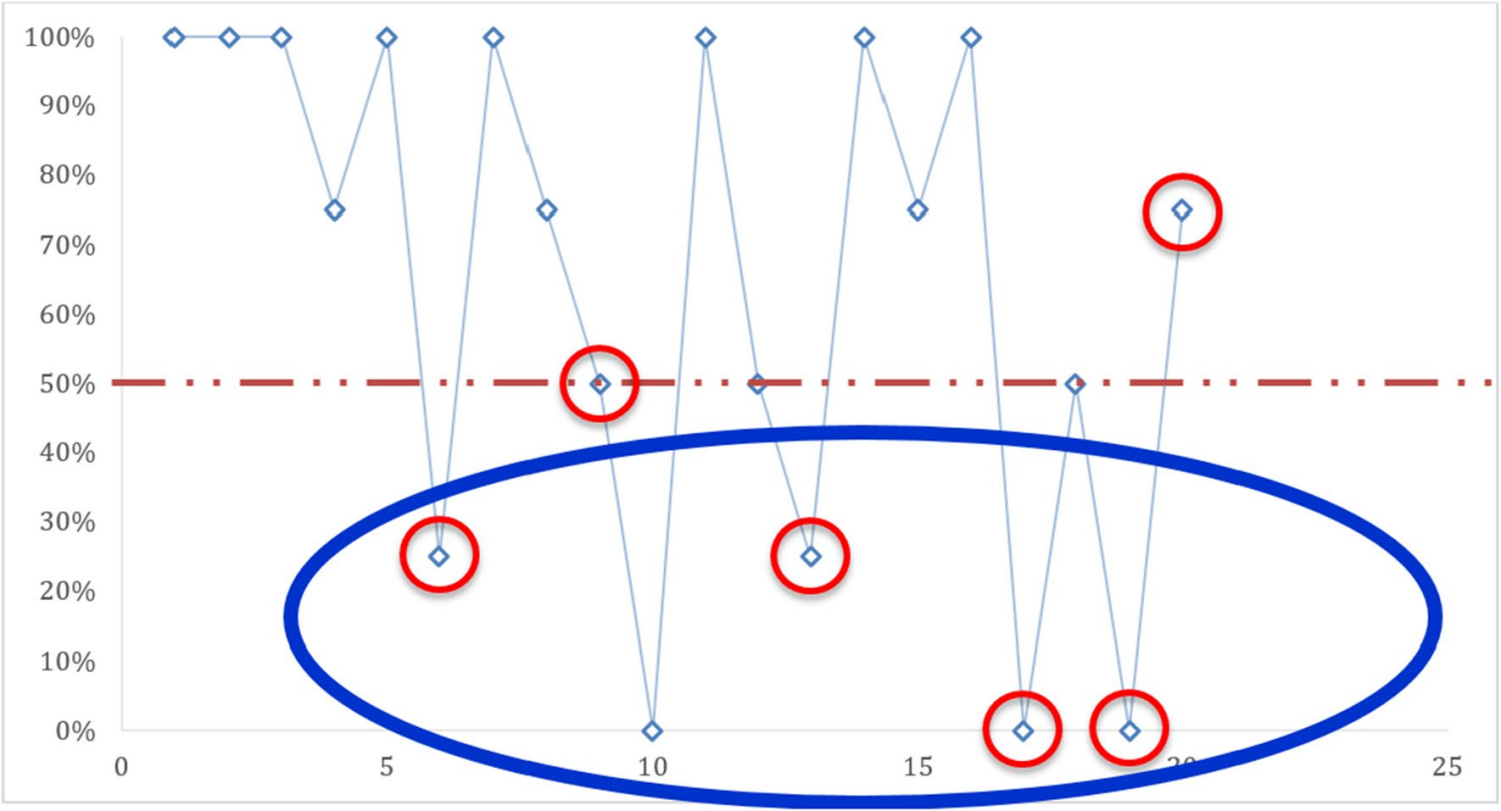
Concordance of results obtained between microscopists on parasite density.  Positive slide for parasite density (PD). Concordance of results obtained (4/6)

**Fig. 3:**
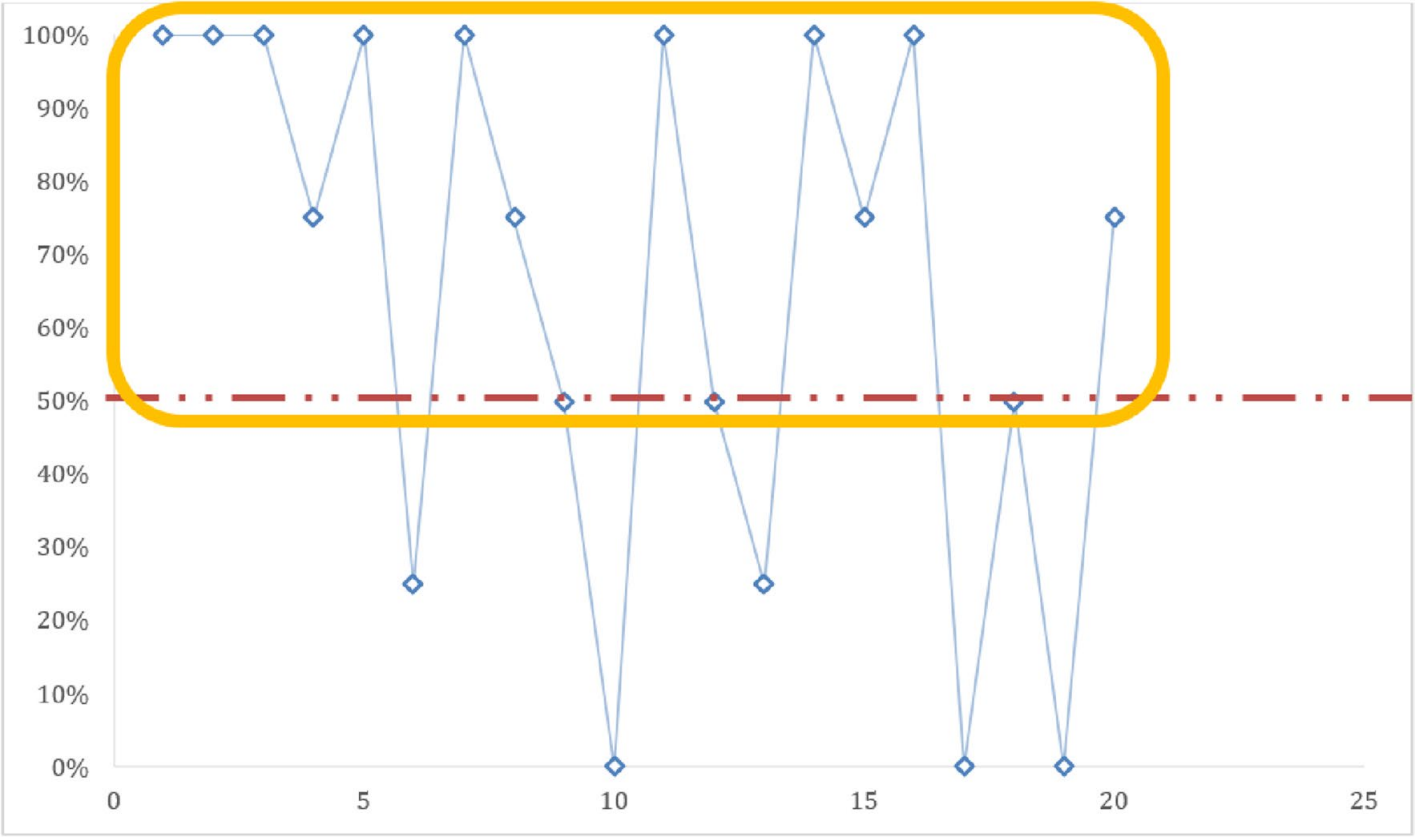
Concordance of results obtained between microscopists on control slide identification.  Concordance of control slide identification

**Fig. 4:**
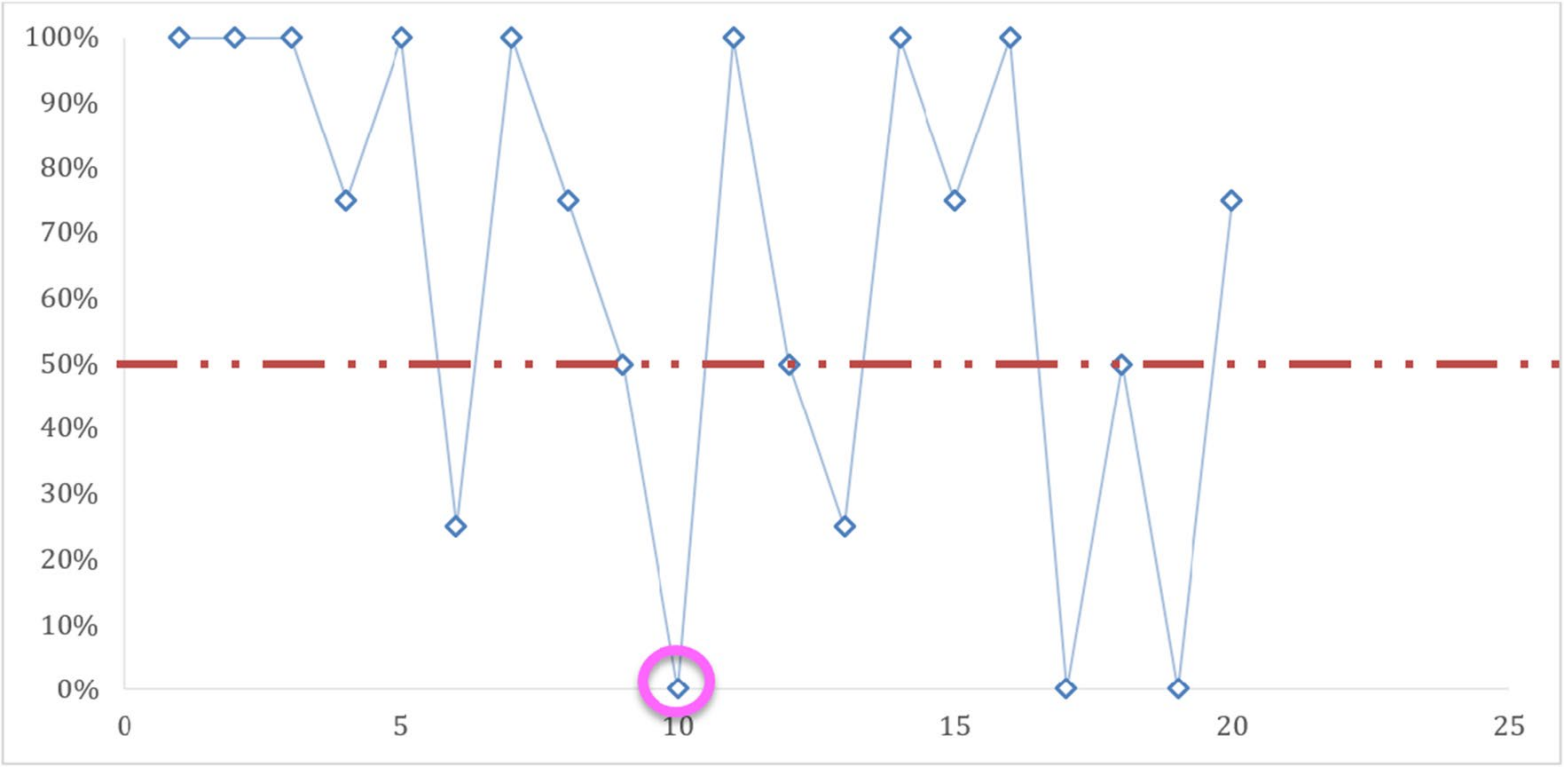
Concordance of results obtained between microscopists on the identification of non-*falciparum* species.  Null concordance on *Plasmodium ovale* (Po) positive slide

During this study, the concordance varied between slides. Thus, on the twenty (20) slides read, a 100% concordance was obtained on the eight (8/20) slides used for determination of positive vs negative slides (Fig. 1). For four (4) of the six (6) parasite density slides, a concordance of less than 50% was noted (Fig. 2). On thirteen (13) out of the fourteen (14) identification slides, a concordance greater than 50% was recorded (Fig. 3). Only one (1) identification slide (the *Plasmodium ovale* slide) met with no concordance from the microscopists (Fig. 4).

### Microscopist skill level: scores compared with references

**Fig. 5:**
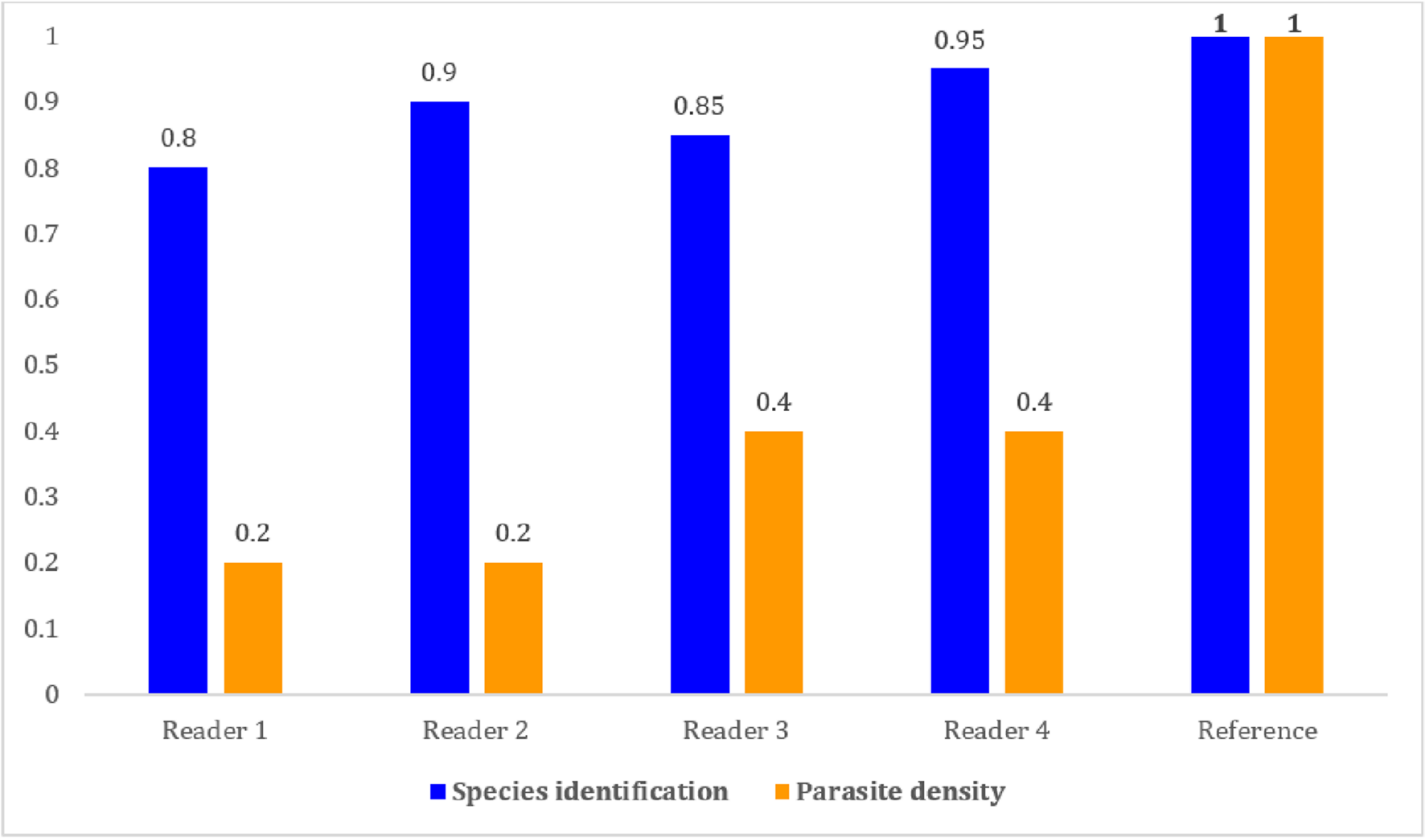
WHO scoring of microscopists [between 0 (lowest) and 1 (highest)]

A score greater than or equal to 80% on the identification of the species compared to the reference and a score ranging from 0.2 to 0.4 on the parasite density were obtained (Fig. 5).

### Precision and accuracy

Microscopists obtained 100% precision and accuracy (Table 3).

**Table 3:** Reliability and accuracy of the different microscopists

Indicators	Reader 1	Reader 2	Reader 3	Reader 4
Precision (%)	100	100	100	100
Accuracy (%)	100	100	100	100

### Microscopist performance

Detailed sensitivity and specificity analysis and predictive validity values

All positive slides as well as the negative ones were correctly identified by the four (4) readers (Table 4).

**Table 4:** Sensitivity, specificity and predictive values

		Presence of parasites		Totals
Reading results		Positives	Negatives	
Slides	Positives	8 (TP)	0 (FN)	8
	Negatives	0 (FP)	6 (TN)	6
Totals		8	6	14

*TP* true positives, *TN* true negatives, *FP* false positives, *FN* false negatives

Determination of sensitivity, specificity, and indices of predictive values

The microscopists gained 100% sensitivity, specificity, and predictive values (Table 5).

**Table 5:** Sensitivity, specificity, and predictive values of the microscopists

Indicators	Reader 1	Reader 2	Reader 3	Reader 4
Sensitivity (%)	100	100	100	100
Specificity (%)	100	100	100	100
Positive predictive value (%)	100	100	100	100
Negative predictive value (%)	100	100	100	100

## Discussion

This work is the beginning of a series of evaluations falling within the framework of the quality approach with a view to the accreditation of the LPM-HALD according to the ISO 15189 Standard. It consisted of the verification of the microscopic method for malaria diagnosis at the LPM- HALD with the aim of supporting the laboratory in this process.

During this study concordance varied between malaria slides. This could be explained by the ability of the microscopists to easily identify *Plasmodium falciparum*. Only the *Plasmodium ovale* (*Po*) identification slide showed discordance. This could be explained by the fact that microscopists are much less familiar with the identification of *P. ovale* which represents less than 2% of the plasmodial species circulating in Senegal as reported by a study carried out in Senegal in 2018, and as notified by the national guide for the biological diagnosis of malaria published in Senegal, in 2015 [6, 14].

In addition, the results of this study showed that four (4) out of six (6) parasite density evaluation slides obtained a concordance of less than 50%. This could be explained by the difficulty that technicians encounter in measuring parasite density. Indeed, this difficulty is even recognized by the WHO in its quality assurance manual where the threshold for parasite density is rather lower (40% and 50% for levels 2 and 1 respectively) compared to the threshold for identification (80% and 90% for levels 2 and 1 respectively). Thus, the WHO and the Senegalese NMCP have recommended a new method for quantifying malaria parasites, requiring the presence of the nucleus, cytoplasm and/or vacuole of the parasite before identifying it and counting it for parasite density [5, 6].

Through the scores produced, our results have verified the skill levels of the microscopists. Indeed, the four (4) microscopists obtained, on species identification, a concordance greater than 80% and the microscopists obtained scores between 0.20 and 0.4 on a scale of 0 to 1 for parasite density reading. This corresponds to level 2 of the WHO which has retained the lower thresholds of 0.4 for Level 2 and 0.5 for Level 1. This level of performance achieved by the LPM-HALD microscopists is considered satisfactory according to the WHO Quality Assurance Manual [5].

These results also verified the fidelity and accuracy of the microscopists. This performance was confirmed by the analysis of sensitivity and specificity and predictive validity values for which microscopists obtained 100% sensitivity, specificity, and predictive values. This means that all the positive slides as well as the negative ones were correctly identified by all four (4) microscopists indicating their performance in detecting the presence or absence of *Plasmodium*. These results exactly correspond to those expected by WHO and Senegal National Malaria Control Programme—[5, 6].

Overall, the performance characteristics measured during this work showed that the LPM-HALD is in line for accreditation according to ISO 15189 standards in relation to the microscopic diagnosis of malaria.

## Conclusion

This study confirms the good performance of malaria microscopy diagnosis at the LPM-HALD. It also highlights difficulties linked to the quantification of parasite density, suggesting the need to strengthen training of microscopists. This study confirms the importance of setting up a quality management system to ensure continuous improvement, with an accessible slide bank to facilitate regular internal and external laboratory quality control to comply with the requirements of ISO 15189 and the NMCP in Senegal.

## Data Availability

The data supporting the findings of this article are included within the article.

## Abbreviations

ACT: Artemisinin-based combination therapy
AQ: Amodiaquin
EEQ: Evaluation Externe de la Qualité
FN: False negative
FP: False positive
HALD: Hôpital Aristide Le Dantec
IPTp: Intermittent preventive treatment in pregnant women
IRS: Indoor residual spray
LLINs: Long-lasting insecticidal nets
LPM: Laboratoire de Parasitologie et Mycologie
NMCP: National Malaria Control Programme
NPV: Negative predictive values
OMS: Organisation mondiale de la sante
PD: Parasite density
PPV: Positive predictive values
RDT: Rapid diagnostic test
Se: Sensitivity
SMC: Seasonal malaria chemoprophylaxis
Sp: Specificity
SP: Sulfadoxine-Pyriméthamine
TN: True negatives
TP: True positives
TS: Thick blood Smear
UCAD: Université Cheikh Anta Diop de Dakar
WHO: World Health Organization
